# Supplementary material for: Dietary Supplementation with Transgenic Camelina sativa Oil Containing 20:5n-3 and 22:6n-3 or Fish Oil Induces Differential Changes in the Transcriptome of CD3+ T Lymphocytes
Source: Nutrients. 2021 Sep 5;13(9):3116. doi: 10.3390/nu13093116 (PMC8466821; doi:10.3390/nu13093116)
Supplement: Supplementary file 1 [file nutrients-13-03116-s001.zip › nutrients-1326144-supplementary.pdf]

Supplementary online material

Supplementary Table S1. qRTPCR primers

Gene target	Primer assay
IL6R	Qiagen Hs_IL6R_1_SG QuantiTect Primer Assay (QT00023660)
CXCL9	Qiagen Hs_CXCL9_1_SG QuantiTect Primer Assay (QT00013461)
PPARα	Qiagen Hs_PPARα_1_SG QuantiTect Primer Assay (QT00017451)
IL17A	Qiagen Hs_IL17A_1_SG QuantiTect Primer Assay (QT00009233)
GPNMB	Qiagen Hs_GPNMB_1_SG QuantiTect Primer Assay (QT00036904)
RPL13A	Primer design reference gene assay (HK-SY-hu)
SDHA	Primer design reference gene assay (HK-SY-hu)

Supplementary Table S2. The effect of dietary supplementation with tCSO or FO in the fatty acid composition of PBMCs from the participants subgroups from which T cells were used in RNAseq analysis. Values are mean ± SEM proportions of total fatty acids (n = 16). There were no significant differences (all P > 0.05) between the whole group and the corresponding RNAseq subgroup in the proportions of any fatty acids by Students unpaired t-test.

	Proportions of total fatty acids (%)			
	FO		t*CS*O	
	Start	End	Start	End
14:0	0.7 ± 0.1	0.5 ± 0.1	0.4 ± 0.1	0.5 ± 0.1
16:0	20.9 ± 1.7	19.4 ± 1.2	19.0 ± 1.9	18.6 ± 1.6
16:1n-7	0.3 ± 0.0	0.2 ± 0.0	0.3 ± 0.0	0.2 ± 0.0
18:0	26.3 ± 1.4	25.5 ± 1.0	28.0 ± 1.6	26.8 ± 1.3
18:1n-9	14.0 ± 1.0	16.7 ± 0.6	14.9 ± 0.9	15.2 ± 0.9
18:1n-7	1.5 ± 0.1	1.4 ± 0.1	1.4 ± 0.1	1.2 ± 0.1
18:2n-6	6.7 ± 0.5	7.1 ± 0.2	7.5 ± 1.0	6.1 ± 0.4
18:3n-6	0.4 ± 0.1	0.2 ± 0.0	0.5 ± 0.1	0.4 ± 0.1
18:3n-3	1.0 ± 0.1	1.1 ± 0.2	0.8 ± 0.1	1.1 ± 0.1
20:0	0.5 ± 0.1	0.3 ± 0.0	0.6 ± 0.2	0.6 ± 0.2
20:1n-9	1.2 ± 0.2	1.0 ± 0.1	1.1 ± 0.1	0.9 ± 0.2
20:2n-6	0.6 ± 0.1	0.3 ± 0.0	0.6 ± 0.1	0.4 ± 0.1
20:3n-6	1.7 ± 0.1	1.6 ± 0.2	1.8 ± 0.2	1.6 ± 0.2
20:4n-6	17.5 ± 1.6	18.4 ± 1.1	16.6 ± 2.0	18.4 ± 1.8
20:5n-3	0.7 ± 0.2	0.9 ± 0.1	0.9 ± 0.2	1.2 ± 0.3
22:5n-3	1.8 ± 0.2	2.2 ± 0.2	1.8 ± 0.3	2.0 ± 0.3
22:6n-3	2.0 ± 0.1	2.5 ± 0.2	2.1 ± 0.3	2.3 ± 0.3

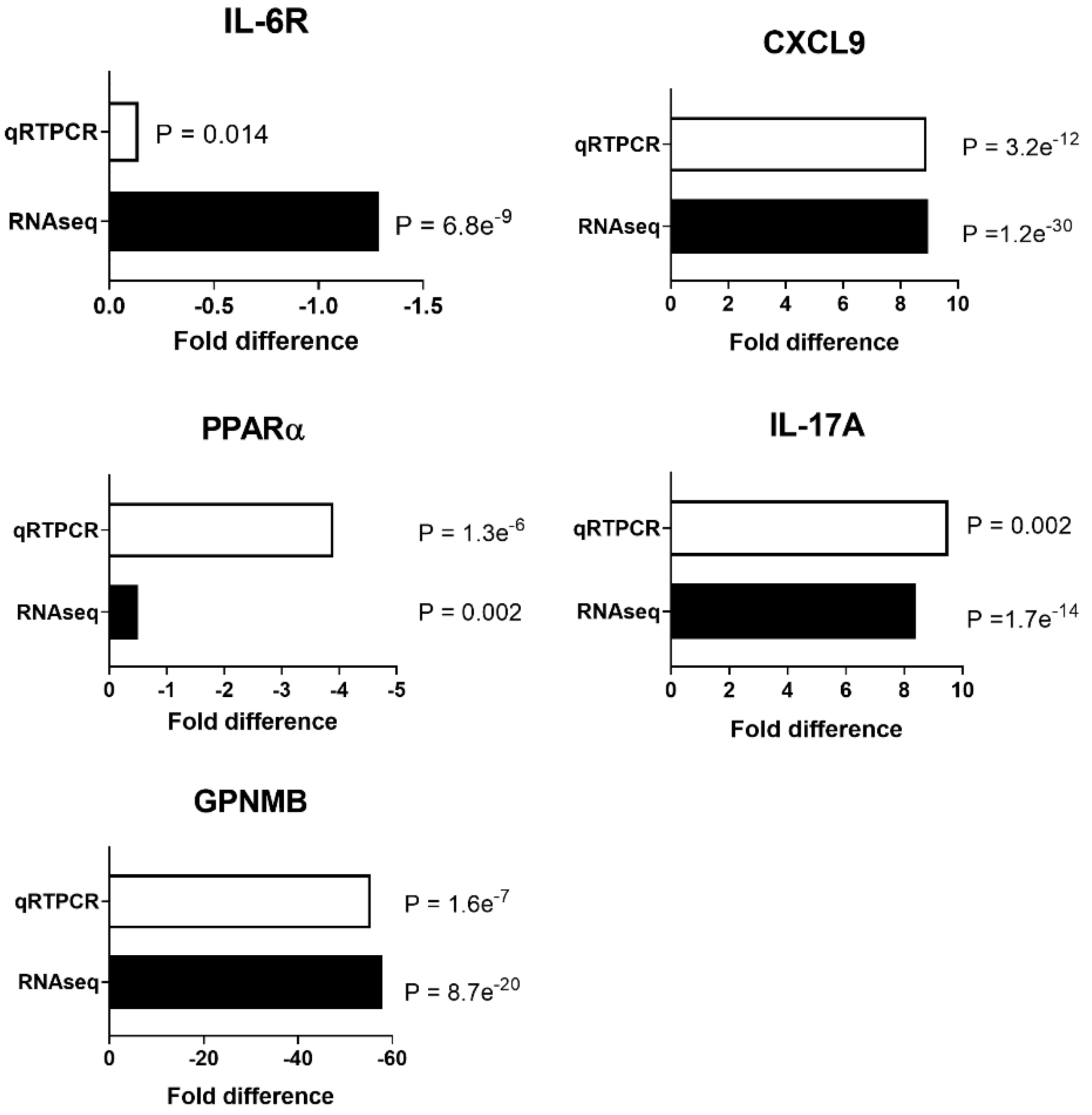

Supplementary Figure S1. qRTPCR validation of RNA sequencing assay. Validation of RNAseq analysis of the effect of mitogen stimulation of T lymphocytes by qRTPCR. Values are fold difference between the level of expression of the respective genes in unstimulated and concanavalin A stimulated T cells collected at baseline (n = 16) prior to dietary supplementation. The difference in mRNA expression measured by qRTPCR was calculated by the ΔΔct method [60]. Statistical analyses were by Students paired t test.
